# DJ-1/FGFR-1 Signaling Pathway Contributes to Sorafenib Resistance in Hepatocellular Carcinoma

**DOI:** 10.1155/2022/2543220

**Published:** 2022-06-20

**Authors:** Xin Chen, Guohua Yang, Xiaohong Guo, Jing Zhang, Wei Sun, Dongbo Liu, Hui Wang, Shunfang Liu

**Affiliations:** ^1^Department of Oncology, Tongji Hospital, Tongji Medical College, Huazhong University of Science and Technology, Wuhan 430030, China; ^2^Department of Medical Genetics, School of Basic Medical Science, Demonstration Center for Experimental Basic Medicine Education, Wuhan University, Wuhan 430071, China; ^3^Department of Medical Biology, School of Basic Medical Sciences, Hubei University of Chinese Medicine, Wuhan 430065, China

## Abstract

Sorafenib is the first-line therapeutic regimen targeting against advanced or metastatic stage of hepatocellular carcinoma (HCC). However, HCC patients at these stages will eventually fail sorafenib treatment due to the drug resistance. At present, molecular mechanisms underlying sorafenib resistance are not completely understood. Our past studies have shown that DJ-1 is upregulated in HCC, while DJ-1 knockdown inhibits HCC xenograft-induced tumor growth and regeneration, implying that DJ-1 may be a potential target in for HCC treatment. However, whether DJ-1 plays a regulatory role between tumor cells and vascular endothelial cells and whether DJ-1 contributes to sorafenib resistance in HCC cells are largely unclear. To address these questions, we have performed a series of experiments in the current study, and we found that (1) DJ-1, one of the molecules secreted from HCC cells, promoted angiogenesis and migration of vascular endothelial cells (i.e., ECDHCC-1), by inducing phosphorylation of fibroblast growth factor receptor-1 (FGFR-1), phosphorylation of mTOR, phosphorylation of ERK, and phosphorylation of STAT3; (2) downregulation of FGFR1 inhibited tube formation and migration of ECDHCC-1 cells stimulated by DJ-1; (3) FGFR1 knockdown attenuated the phosphorylation of FGFR1 and impaired the activity of Akt, ERK, and STAT3 signals induced by DJ-1 in ECDHCC-1 cells; (4) knocking down FGFR1 led to the elevated expression of proapoptotic molecules but deceased level of antiapoptotic molecules in sorafenib-resistant HCC cells; and (5) Downregulation of FGFR1 suppressed tumor growth and angiogenesis of sorafenib-resistant HCC cells *in vivo*. Altogether, our results hinted that DJ-1 plays vital roles in tumor microenvironment in HCC development, and DJ-1/FGFR1 signaling pathway may be a therapeutic target for overcoming sorafenib resistance in treating HCC patients at the late stage.

## 1. Introduction

Hepatocellular carcinoma (HCC) is the predominant type of liver cancer. At present, partial hepatectomy and liver transplantation offer the best prognosis for HCC patients when the tumor is <5 cm in diameter, limited to one lobe of the liver, without invasion of liver vasculature, and liver function is well preserved [1]. Unfortunately, a high postoperative tumor recurrence rate significantly decreases long-term survival. Owing to the invasive growth and late symptom presentation, most patients are diagnosed at advanced stages and are not eligible for surgery [2]. Sorafenib, an orally available tyrosine kinase inhibitor, is the first-line drug against advanced HCC since 2008, which significantly improved the overall survival of unresectable HCC patients. However, the promising therapy has demonstrated limited clinical efficacy, typically only extending patients' survival by about 3 months. Some patients with HCC initially respond well to sorafenib but will eventually fail due to cancer progression, indicating that there is an acquired resistance to sorafenib in HCC [3]. Up to now, the mechanism responsible for this acquired resistance of HCC to sorafenib is unclear, and we propose a possible means by which it is achieved.

As a novel oncogene, protein deglycase DJ-1 is a 20 kDa protein which is abundantly expressed in more than 22 human tissues, including the liver and vascular endothelium [4]. DJ-1 is associated with multiple biological functions, such as transcriptional regulation, chaperone activity regulation, protease function regulation, and mitochondrial regulation [5]. Meanwhile, it has been reported that DJ-1 is one of the regulators in tumorigenesis, invasion, and metastasis in various cancers, including HCC [6]. Our past research found that DJ-1 was upregulated in HCC. In addition, downregulation of DJ-1 resulted in decreased proliferation, adhesion and invasion of HepG2 cells *in vitro*, and inhibited the growth of HepG2-induced tumor *in vivo*, which implies its crucial role for DJ-1 in the oncogenesis of HCC [7, 8]. Meanwhile, DJ-1 has been found to promote angiogenesis during osteogenesis and has been identified as a communicating factor between osteoblasts and endothelial cells [9]. As it is well known that antiapoptosis of tumor cells and angiogenesis of vascular endothelial cells are both involved in sorafenib resistance, inhibition of angiogenesis in tumor is expected to overcome drug resistance to HCC. Whether DJ-1 plays a crucial role in angiogenesis of HCC vascular endothelial cells and acts as a cross-talk regulatory role between tumor cells and vascular endothelial cells in HCC sorafenib resistance is largely unclear.

Fibroblast growth factor receptor 1 (FGFR1), a tyrosine kinase receptor, is the predominant type of FGFR and plays a key role in promoting angiogenesis in endothelial cells. FGF induced the phosphorylation of FGFR1 and activated downstream signaling molecules, such as Src kinase, focal adhesion kinase (FAK), and extracellular signal-regulated kinase 1/2 (ERK1/2), which have been implicated in endothelial cell differentiation [10]. Meanwhile, sorafenib suppresses tumor angiogenesis and proliferation by inhibiting multikinases such as the serine/threonine kinase family (e.g., Raf-1, PI3K/Akt, ERK1/2, and STAT3) and the receptor tyrosine kinase family (e.g., FGFR, VEGFR, and PDGFR) [3]. Furthermore, the acquired resistance to sorafenib in HCC has been linked to the activation of FGF/FGFR1 signaling pathway [11]. Importantly, it has been reported that DJ-1 can induce the phosphorylation of FGFR1 and activate the FAK and ERK1/2 signal pathway, which result in the stimulation of migration and capillary formation of vascular endothelium [9]. Therefore, it is reasonable to suspect that the DJ-1/FGFR1 signaling pathway might contribute to sorafenib resistance in HCC, and the inhibition of DJ-1/FGFR1 pathway will benefit a subset of sorafenib resistant patients with high expression of DJ-1.

Here, DJ-1 was targeted as a cross-talk regulator between HCC cells and vascular endothelial cells, and the proangiogenic effect of DJ-1/FGFR1 signaling in ECDHCC cells was investigated. We also explored the underlying mechanisms on the reversal effect of sorafenib resistance in HCC cells by downregulating FGFR1.

## 2. Materials and Methods

### 2.1. Cell Culture and Establishment of Sorafenib Resistance in HCC Cell Lines

The human hepatocellular carcinoma cell lines (HepG2 and HUH-7), endothelial cells derived from hepatocellular carcinoma (ECDHCC-1), THLE-2, and L02 cells derived from healthy liver cells were all obtained from American Type Culture Collection (ATCC; Manassas, VA, USA). Cells were cultured at 37°C with 5% CO_2_ in Dulbecco's modified Eagle's medium (DMEM) high-glucose medium containing 10% fetal serum (Gibco-Life Technologies, Carlsbad, CA, USA) and 1% penicillin and streptomycin. To establish the sorafenib-resistant HCC cell lines, the half-maximal inhibitory concentration (IC_50_) of HCC cells to sorafenib was initially determined by incubating cells with different concentrations of sorafenib in 96-well plates, and cell viability was measured 3 days later as described below. The cells were cultured in 6-well plates at 1 × 10^4^ cells/well and incubated with sorafenib at a concentration just below their respective IC_50_. The concentration of sorafenib was slowly increased by 0.25 *μ*M per week. After 6 to 7 months, sorafenib-resistant cell lines were obtained and continuously maintained in the presence of sorafenib. The resistance index (RI) was calculated as the ratio of IC_50_ of sorafenib resistant cell line to IC_50_ of corresponding control cell line.

### 2.2. ELISA Assay of DJ-1

DJ-1 level in the culture medium was assessed by enzyme-linked immunosorbent assay using a commercially available human protein DJ-1 (PARK7) ELISA kit (#CSB-E12024h, CUSABIO, Wuhan, China) according to the manufacture's protocol.

### 2.3. Expression and Purification of His-Tagged DJ-1 Protein

Expression and purification of recombinant DJ-1 were carried out using the pET28a vector according to the supplier's protocols (EMD Millipore, Darmstadt, Germany). DJ-1 was cloned into the pET28a vector and sequenced. The DJ-1 construct was transformed into Escherichia coli BL21 (DE3) for expression of His-tagged DJ-1 (His DJ-1), and recombinant protein was purified on nickel-NTA agarose columns. Finally, the concentration and purity of His-tagged DJ-1 protein were determined using the Bradford assay (Bio-Rad), and the production was verified by western blotting analysis with anti-His tag and anti-DJ-1 specific antibody, respectively.

### 2.4. Construction of FGFR1 shRNA Expression Plasmid

The pLKO.1 lentiviral plasmid was selected to construct the shFGFR vector. Empty vector was used as a control group. To generate lentiviruses, HEK 293T cells were cotransfected with pLKO-shFGFR1 plasmid, psPAX2 packaging plasmid, and pMD2.G envelop plasmid using Lipofectamine 3000 (Invitrogen). Transduction of pLKO-shFGFR1 lentivirus and stable FGFR1 knock down was conducted according to the manufacturer's protocols. Short hairpin RNAs (shRNA) targeting FGFR1 (shFGFR1) were detailed as follows: FGFR1-Sh1: 5′- CCA CAG AAT TGG AGG CTA CAA-3′, FGFR1-Sh2: 5′-GAT GGC ACC CGA GGC ATT ATT-3′, FGFR1-Sh3: 5′-TGC CAC CTG GAG CAT CAT AAT-3′.

### 2.5. Cell Proliferation Assay of CCK8

Cell proliferation was evaluated through a colorimetric assay by using cell counting kit-8 (CCK-8, GLPBIO, USA) reagent following the manufacturer's protocol. Briefly, ECDHCC cells were seeded into 96-well plates at a density of 5 × 10^3^ cells/well in a medium with 10% FBS and allowed to adhere for 24 h. Next, cells were cultured in a medium with 1% FBS in the presence or absence of indicated medium or reagent (Sigma-Aldrich, St. Louis, MO, USA) for a further 24 h and/or 48 h. Afterwards, 10 *μ*L of CCK-8 reagent was added and maintained for 3 h. A microplate reader (Multiskan MK3, Thermo, Waltham, MA) was used to measure the optical density of 450 nm and 630 nm.

### 2.6. Tube Formation Assay

Matrigel (# K905-50, Biovision, Milpitas, USA) was dissolved at 4°C overnight, and 48-well plates were prepared with 100 *μ*L Matrigel in each well after coating and incubating at 37°C overnight. ECDHCC cells (1 × 10^5^ cells/well) were plated and incubated for 6 h at 37°C. ECDHCC cell tube formation was assessed with a photomicroscope, where tube branches and total tube length were calculated using the angiogenesis plugin of Image J software (NIH, Bethesda, MD, USA).

### 2.7. Wound Healing Assays

Migration activity was measured using wound healing assays. ECDHCC cells were seeded into 6-well plates. After the cell density reached 80% confluence, a wound was created by scratching the cell monolayer with a 200 *μ*L pipette tip. Wound healing was monitored, and the migration distance was imaged at different time points. Experiments were repeated in triplicate.

### 2.8. Flow Cytometry Assessment of Cell Apoptosis

Cell apoptosis was analyzed by annexin-V/PI. In brief, cells were cultured in a 6-well plate at a density of 1 × 10^6^ cells/well and incubated with indicating reagent for 24 h. Cells were then trypsinized and harvested by centrifugation, washed with cold PBS, and resuspended in 200 *μ*L binding buffer. Then, cells were stained with annexin-V-fluorescein isothiocyanate (FITC, 0.5 *μ*g/mL) and PI (50 *μ*g/mL) in the dark for 15 min and analyzed using a flow cytometer (FACScan, Becton-Dickinson, NJ, USA).

### 2.9. Co-Immunoprecipitation (Co-IP)

Cells were washed twice in ice-cold PBS, harvested, and lysed with RIPA lysis buffer (#P0013D, Beyotime, Wuhan, China) for immunoprecipitation experiments. For each sample, 1.5 mg of protein was incubated with protein A+G agarose fast flow (#P2028, Beyotime, Wuhan, China) and anti-DJ-1 rabbit pAb (#382793, 1 : 1000, ZEN, Wuhan, China). After an overnight incubation at 4°C, the beads were washed, and the final pellet was suspended in RIPA buffer. Bound proteins were eluted from the beads by heating and centrifugation and then analyzed by western blotting analysis.

### 2.10. Quantitative Real-Time Polymerase Chain Reaction (qRT-PCR)

Total RNA was extracted by using TRIzol reagent (Takara, Japan). The reverse transcription reaction was performed using 1 *μ*g of total RNA that was reverse transcribed into cDNA using oligo(dT) primers. Real-time PCR was carried out using the SYBR Premix Ex Taq (Takara, Japan) according to the manufacturer's protocol with the ABI-7500 (ABI, USA). Briefly, 2 *μ*L cDNA template was added to each 20 *μ*L reaction with sequence-specific primers: FGFR1 F: 5′- CGG GAC ATT CAC all ATC GA-3′, R: 5′-CCG CCC AGA GTG AAG ATC TC-3′; *β*-Actin F: 5′- TGA CGT GGA CAT CCG CAA AG-3′, R: 5′- CTG GAA GGT GGA CAG CGA GG-3′. *β*-Actin was used as an endogenous control to normalize expression data. The cycling conditions comprised initial 10 min polymerase activation at 95°C, followed by 40 cycles at 95°C for 15 s, 60°C for 60 s, and 72°C for 15 min. The FGFR1 transcript levels were quantified using 2^−*ΔΔ*CT^ method [12].

### 2.11. Western Blotting Analysis

Total protein was extracted from cells using the RIPA buffer (PPLYGEN, C1053, China) supplemented with protease inhibitor cocktail (Roche, Switzerland). Western blotting was performed by SDS-PAGE gels and transferred onto polyvinylidene fluoride (PVDF) membranes. After blocking with 5% nonfat milk, membranes were separately probed with primary antibodies. Our primary antibodies are listed below: p-FGFR1-Tyr653/654 mouse mAb (#3476, 1 : 500, CST, USA), FGFR1 mouse mAb (#60325-1-Ig, 1 : 1000, Proteintech, USA), p-Akt-S473 rabbit pAb (#AP0140, 1 : 1000, ABclonal, China), Akt rabbit pAb (#11016, 1 : 1000, ABclonal, China), p-mTOR-S2448 rabbit pAb (#AP0094, 1 : 1000, ABclonal, China), mTOR rabbit pAb (#20657-1-AP, 1 : 1000, Proteintech, USA), p-ERK1/2 (ERK1-T202/Y204+ERK2-T185/Y187) rabbit pAb (#AP0472, 1 : 1000, ABclonal, China), ERK1/2 rabbit pAb (#16443-1-AP, 1 : 1000, Proteintech, USA), p-STAT3- Tyr705 rabbit pAb (#381552, 1 : 1000, ZEN, China), STAT3 rabbit pAb (#A11216, 1 : 1000, ABclonal, China), cleaved caspase 3 rabbit pAb (#A2156, 1 : 1000, ABclonal, China), cleaved caspase 9 rabbit pAb (#A2636, 1 : 1000, ABclonal, China), Bax rabbit pAb (#50599-2-Ig, 1 : 1000, Proteintech, USA), Bcl-2 rabbit pAb (#12789-1-AP, 1 : 1000, Proteintech, USA), and GAPDH rabbit mAb (#2118S, 1 : 1000, CST, USA). After incubation at 4°C overnight, membranes were washed and incubated with appropriate secondary antibody (1 : 3000, ABclonal, China). The proteins of interest were detected by SuperSignalTM West Pico PLUS Chemiluminescent Substrate (Thermo, USA) using a Gel Imaging System (GE Biosciences, Buckinghamshire, England).

### 2.12. Animal Studies

Nude male mice (5-week-old) were purchased from Beijing Vital River Laboratory Animal Technology Co., Ltd. Our animal studies were performed under the protocols approved by the IACUC of Huazhong University of Science and Technology. Basic procedures have been described previously [13]. In brief, we chose HUH-7/R cell line as our model and design the in vivo experiments with 3 groups: (1) HUH-7/R+control; (2) HUH-7/R+sorafenib+DJ-1; and (3) HUH-7/R+sorafenib+DJ-1+shFGFR1. Cells (5 × 10^6^ cells/injection) were injected subcutaneously into nude mice. We measured tumor growth twice to three times weekly in two dimensions using a digital caliper, and tumor volume was calculated using 1/2 (length × width^2^). At the end of the experiments, xenograft tumors were harvested, and tumor weight, incidence, and tumor images were recorded.

### 2.13. Chicken Chorioallantoic Membrane (CAM) Model

Basic procedures were performed as previously described [14]. In brief, we used the fertilized chicken eggs that were incubated at 37°C, and the CAM membrane was scored on ~day 10 of embryonic development to remove the top layer of the CAM epithelium. 2 × 10^6^ HCC cells (i.e., HUH7 and HUH7/R) were mixed with 2 × 10^6^ ECDHCC-1 cells at the ratio of 1 : 1, which were treated and grouped as follows: (1) mixed cells+control; (2) mixed cells+DJ-1; (3) mixed cells+sorafenib; (4) mixed cells+sorafenib+DJ-1; and (5) mixed cells+sorafenib+DJ-1+shFGFR1. We added finally manipulated cells onto the scored CAM. Chicken embryos were then incubated and maintained at 37°C during the experiments.

### 2.14. Statistical Analysis

Data are presented as mean ± SD. Differences were performed using the student's *t*-test for two groups or one-way ANOVA for multiple groups with Bonferroni's post hoc test by use of GraphPad 5.0 software (GraphPad Prism, CA, USA). In all cases, *P* < 0.05 was considered significant.

## 3. Results

### 3.1. HCC Culture Medium Promotes Angiogenesis and Migration of ECDHCC-1 Cells

**Figure 1:**
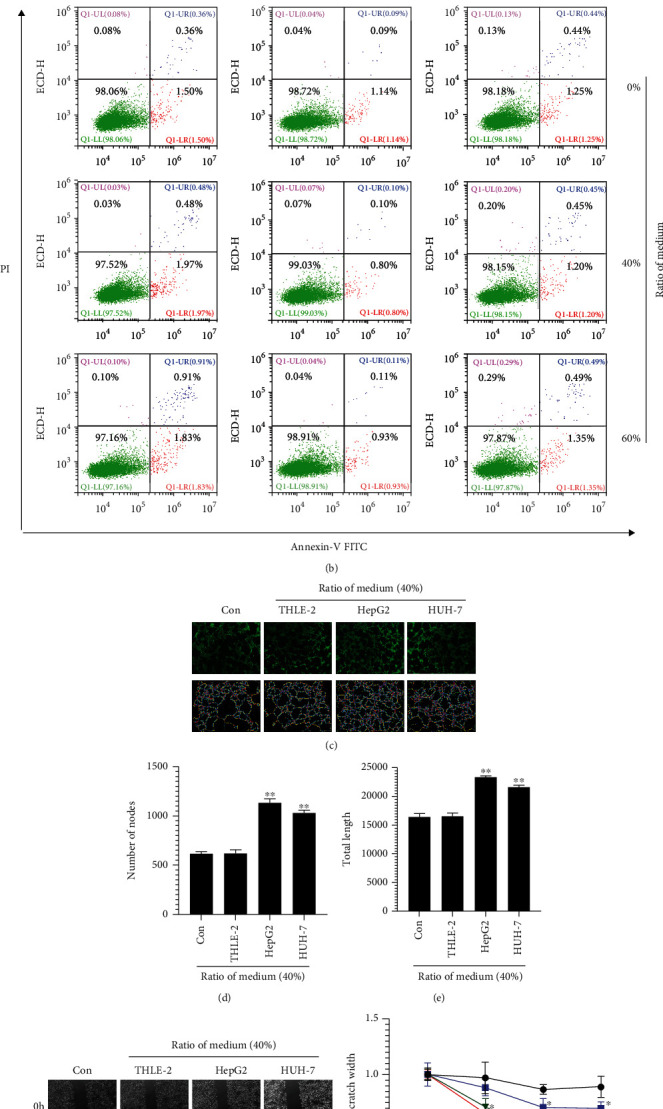
Effect of HCC culture medium on biological characteristics of ECDHCC-1 cells. (a and b) Cell viability (a) and cell apoptosis (b) of ECDHCC-1 cells were checked after incubation with HepG2-, HUH-7-, or THLE-2-conditioned media at the indicated ratio to ECDHCC-1 medium. (c–e) Fluorescence microscopy imaging of tube formation (c) and quantification of the number of nodes (d) and total tube length (e) by using tube formation assay utilizing the indicated medium with a 40% ratio to ECDHCC-1 medium were analyzed. (f and g) Wound healing (f) and quantification of the relative scratch width (g) of ECDHCC-1 cells treated with the indicated medium with a 40% ratio to ECDHCC-1 medium were performed. Con: Control group. ^∗^*P* < 0.05 and ^∗∗^*P* < 0.01 versus Con. (original magnifications: ×200 for fluorescence).

To test how HCC cells interact with vascular endothelial cells, we first collected the culture medium from HepG2, HUH-7, and THLE-2 cells, which was added to the culture medium of ECDHCC-1 cells at different ratios. CCK8 assays showed that ECDHCC-1 has a better cellular growth behavior when HCC or THLE-2 cell culture medium was at 10-60% ratio of ECDHCC-1 cell culture medium (Figure 1(a)), and no apoptotic effect was observed in each culture medium at 40% and 60% ratio (Figure 1(b)). Thus, the conditioned medium was used at a ratio of 40% in the tube formation and wound healing experiments. Our results showed that the numbers of nodes and total tube length increased in the HepG2- and HUH-7-treated group (Figures 1(c)–1(e)), and the migration of ECDHCC-1 cells was increased in the HepG2 and HUH-7 group at 12, 24, and 36 h. There was also a slight increased trend observed in the THLE-2 group at 24 and 36 h (Figures 1(f) and 1(g)). These results suggested that secretory factors in the culture medium of HCC cells may promote angiogenesis and migration of vascular endothelial cells.

### 3.2. DJ-1 Could Significantly Induced Angiogenesis and Migration of ECDHCC-1 Cells

**Figure 2:**
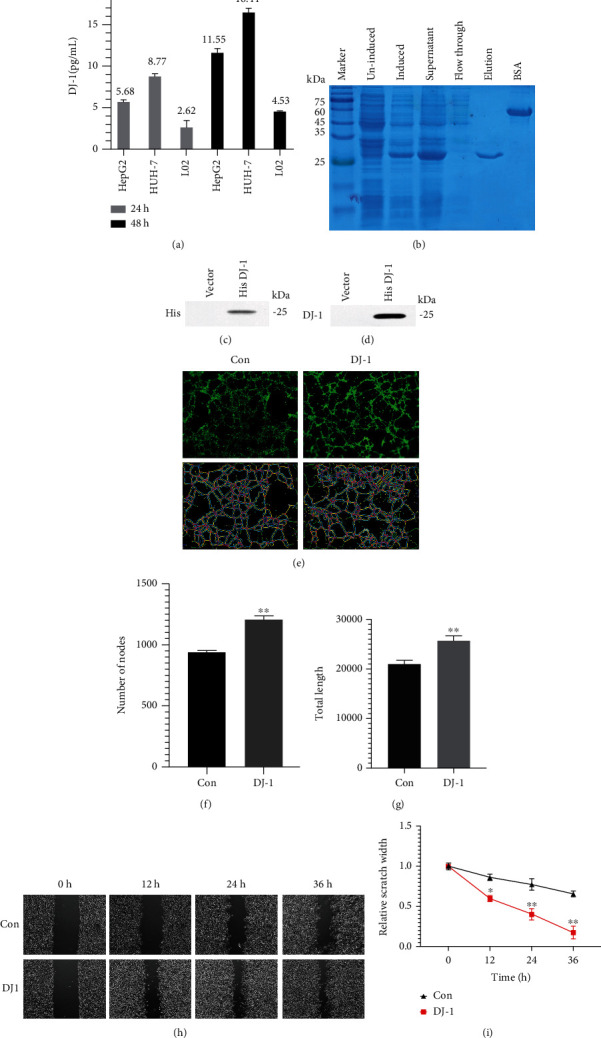
DJ-1 induced of angiogenesis and migration in ECDHCC-1 cells. (a) DJ-1 secretion in HCC cells. HepG2 and HUH-7 cells were seeded for culture medium (CM) collection. The CM was harvested and examined by ELISA for DJ-1 expression after 24 and 48 h. (b) Purification of DJ-1 as indicated in the elution lane. (c and d) Product verification of DJ-1 by western blotting analysis with anti-His tag (c) and anti-DJ-1 (d) specific antibody was performed, respectively. (e–g) Fluorescence microscopy imaging of capillary tube formation (e) and quantification of DJ-1 induced tube nodes (f) and tube length (g) using tube formation assay were conducted. (h and i) Wound healing image (h) and quantification of the relative scratch width (i) of ECDHCC-1 cells induced by DJ-1 were analyzed. Con: Control group. ^∗^*P* < 0.05 and ^∗∗^*P* < 0.01 versus Con. (original magnifications: ×200 for fluorescence).

Previous studies have reported that DJ-1 is a soluble protein and may play important roles in neuronal maintenance and oxidative stress as well as cancer progression [15, 16]. To investigate if DJ-1 is one of the secretory factors in HCC cell culture medium and functions to help endothelial cells for their angiogenesis and migration, DJ-1 level in culture medium was assessed by ELISA, and the results showed that DJ-1 was secreted in HepG2, HUH-7, and L02 cells at an average of 5.68, 8.77, and 2.62 pg/mL in 24 h, respectively, and DJ-1 level increased to an average of 11.55, 16.44, and 4.53 pg/mL at 48 h, respectively (Figure 2(a)). Subsequently, the DJ-1 gene was cloned and expressed as a protein with the molecular weight at about 25 kDa. DJ-1 was then purified at a concentration of 0.758 mg/mL at a purity of above 85%, which was verified by western blotting of anti-His tag and anti-DJ-1 specific antibodies (Figures 2(b)–2(d)). Then, cell viability of ECDHCC-1 was assayed by CCK8 assay after treatment by purified DJ-1 at 5, 10, 15, and 20 *μ*g/mL, respectively, and no significant difference was detected between the treated and untreated groups (data not shown). Thus, purified DJ-1 at the concentration of 15 *μ*g/mL was used in the following experiments. Interestingly, the results of the tube formation assay revealed a significant induction of angiogenesis as determined by the increased number of nodes and total tube length (Figures 2(e)–2(g). Meanwhile, cell migration was also enhanced significantly after DJ-1 treatment (Figures 2(h) and 2(i)). These results suggested that DJ-1 might function as a proangiogenic factor and migration facilitator *in vitro*.

### 3.3. The Effect of DJ-1 Is Involved in the Activation of the Akt/mTOR, ERK1/2, and STAT3 Signaling Pathways, Probably Mediated via FGFR1

**Figure 3:**
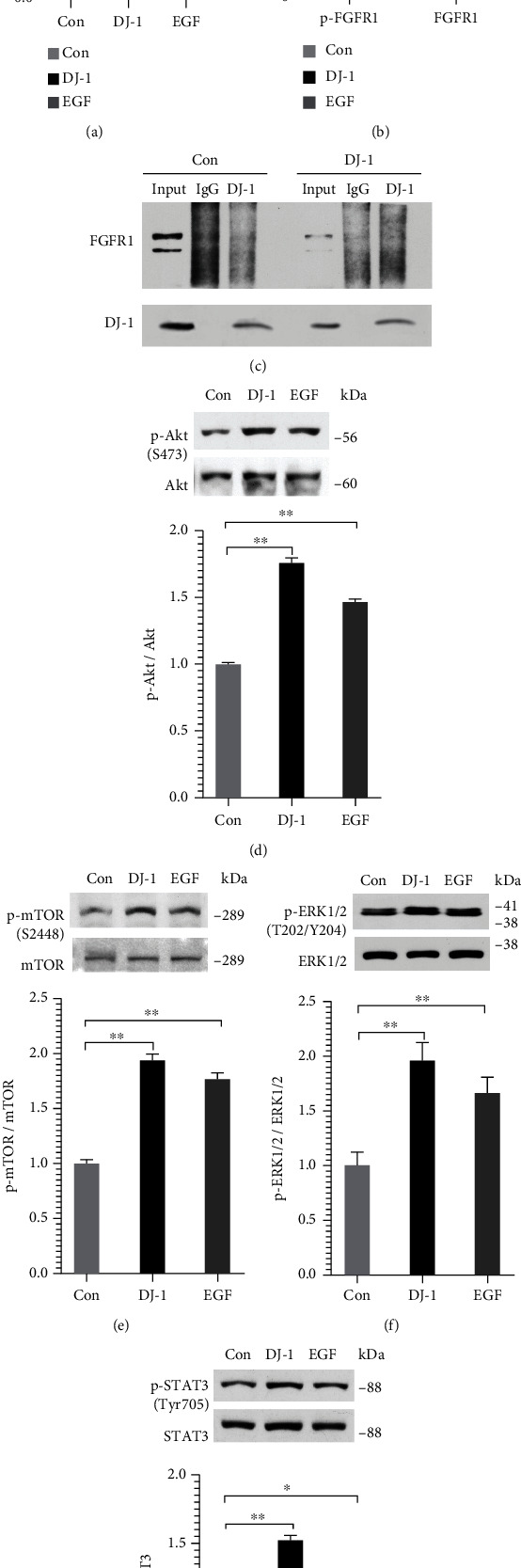
DJ-1 induced FGFR1 phosphorylation and activated the Akt, ERK, and STAT signaling pathways in ECDHCC-1 cells. (a and b) Cells were treated with DJ-1 (15 *μ*g/mL) for 24 h, and then the mRNA expression of FGFR1 (a) and protein expression of phosphorylated FGFR1 (p-FGFR1) and total FGFR1 (b) were detected by qRT-PCR and western blotting analysis, respectively. The inducing activity of DJ-1 was compared with that of EGF (20 ng/mL). (c) Co-immunoprecipitation assay for FGFR1 and DJ-1 was performed. (d–g) Western blotting analysis of Akt, mTOR, ERK, and STAT3 phosphorylation was conducted. The inducing activity of DJ-1 was compared with that of EGF (20 ng/mL). ^∗^*P* < 0.05 and ^∗∗^*P* < 0.01 versus Con.

DJ-1 induces FGFR1 signaling, which results in angiogenesis in endothelial cells [9]. To explore whether the effect of DJ-1 on angiogenesis of ECDHCC-1 cells was mediated by FGFR1 in our study, the mRNA and protein levels of FGFR1 were detected by qRT-PCR and western blotting, respectively. Our results showed that there was no significant difference in FGFR1 mRNA levels between the control and DJ-1 treatment groups (Figure 3(a)). However, there was a significant upregulation of protein expression of both p-FGFR1 and total FGFR1 after DJ-1 treatment (Figure 3(b)). Subsequently, a co-immunoprecipitation assay was carried out, but the results showed that there was no direct interaction between DJ-1 and FGFR1 (Figure 3(c)). Moreover, western blotting of the downstream angiogenesis and migration signaling pathways showed that DJ-1 activated Akt/mTOR, ERK1/2, and STAT3 signaling pathways significantly (Figures 3(d)–3(g)). These results suggested that the effect of DJ-1 on ECDHCC-1 involved activation of Akt/mTOR, ERK1/2, and STAT3 signaling pathway, which might be mediated, at least partially, by FGFR1.

### 3.4. Downregulation of FGFR1 Inhibited the Effect of DJ-1 through Inactivation of Akt/mTOR, ERK1/2, and STAT3 Signaling Pathways

**Figure 4:**
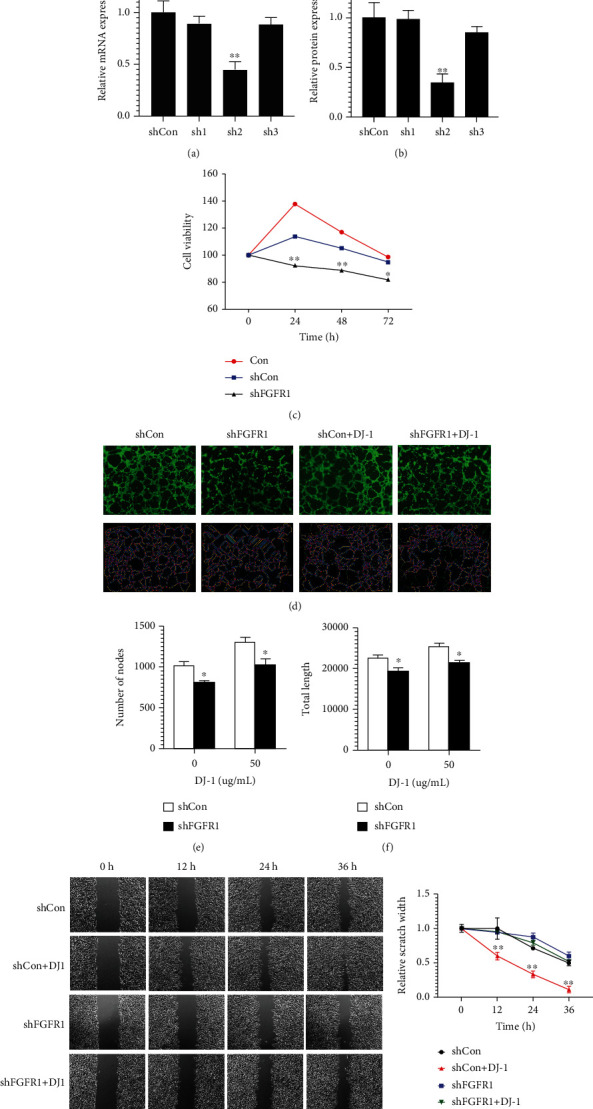
Downregulation of FGFR1 inhibited tube formation and migration of ECDHCC-1 cells stimulated by DJ-1. (a) and (b) Knockdown of FGFR1 by shRNA was confirmed by qRT-PCR and western blotting analysis. (c) Cell viability was analyzed by CCK8. (d–f) The effect of FGFR1 knockdown on DJ-1 inducing angiogenesis on EDCHCC-1 cells was assessed for tube formation assay (d), the number of nodes (e), and total length (f) of capillary tube. *n* = 3 for all groups. (g) and (h) The effect of FGFR1 knockdown on DJ-1 inducing migration on EDCHCC-1 cells was examined. Photographs (×20) were taken at 12, 24, and 36 h, respectively, after treatment with 15 *μ*g/mL DJ-1 (g). Relative scratch width was quantified by image J software (h). ^∗^*P* < 0.05 and ^∗∗^*P* < 0.01 versus Con. (original magnifications: ×200 for fluorescence).

**Figure 5:**
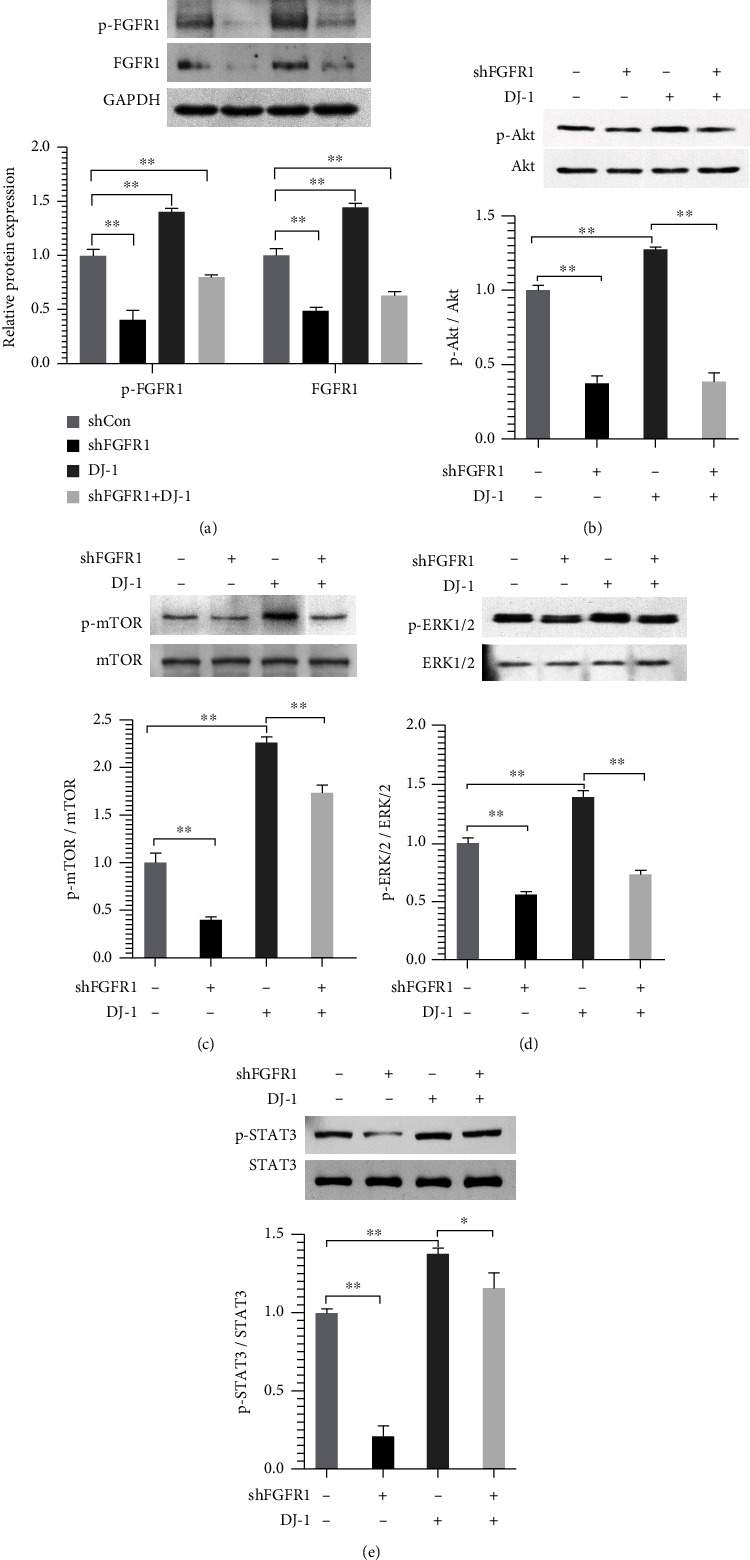
Downregulation of FGFR1 attenuated the phosphorylation of FGFR1 and reversed the activity of Akt, ERK, and STAT3 signal induced by DJ-1. Cells with stable knockdown of FGFR1 were treated with or without DJ-1 (15 *μ*g/mL) for 24 h, and then, the protein expression of p-FGFR1/FGFR1 (a), p-Akt/Akt (b), p-mTOR/mTOR (c), p-ERK1/2/ERK1/2 (d), and p-STAT3/STAT3 (e) was examined by western blotting, respectively. The empty vector group was used as the control (Con). ^∗^*P* < 0.05 and ^∗∗^*P* < 0.01 versus Con.

Furthermore, three FGFR1 shRNA plasmids named sh1, sh2, and sh3 were transfected into ECDHCC-1 cells, respectively. qRT-PCR and western blotting both verified effective downregulation of FGFR1 by the sh2 plasmid (Figures 4(a) and 4(b)). The following experiments were carried out using sh2, which was referred to shFGFR1. CCK8 assay showed that cell viability was decreased after downregulation of FGFR1 (Figure 4(c)). The increased number of nodes and total tube length, as well as migration ability observed after DJ-1 treatment, was all attenuated by knockdown of FGFR1 (Figures 4(d)–4(h)). Interestingly, while DJ-1 induced upregulation of FGFR1, phosphorylation of AKT/mTOR, ERK1/2, and STAT3 was also reversed after knockdown of FGFR1 (Figures 5(a)–5(e)). These results suggested that the DJ-1 induced angiogenesis and migration in ECDHCC-1, probably mediated via FGFR1.

### 3.5. Downregulation of FGFR1 Could Impact on the Survival of Sorafenib-Resistant HCC Cells via Regulating Apoptosis-Associated Molecules

**Figure 6:**
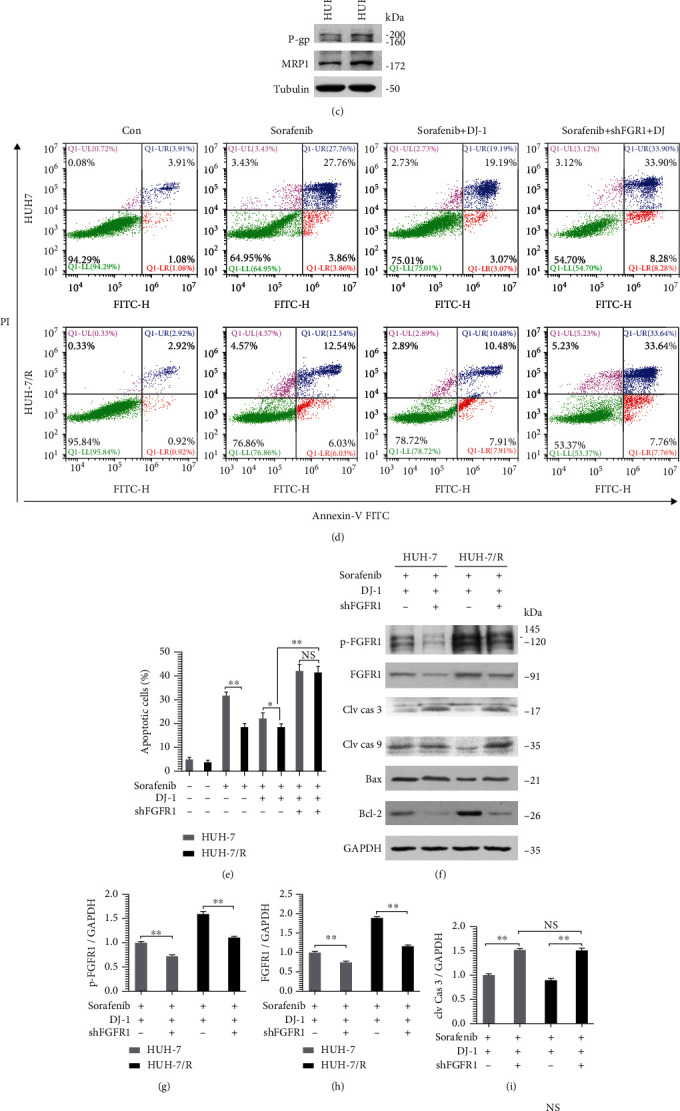
Downregulation of FGFR1 affected the apoptosis of sorafenib-resistant HCC cells. (a) Cell viability of THLE-2, HepG2, and HUH-7 after treatment with indicated concentration of sorafenib was assessed by CCK8 assay, and half-maximal inhibitory concentration (IC_50_) was calculated with a linear fit. (b) Sorafenib-resistant HUH-7 cell line (HUH-7/R) was constructed, and the cell viability was assessed to define the resistance index (RI) of the HUH-7/R cell line. (c) Western blotting analysis of multidrug resistance marker of P-gp and MRP1 was detected. (d and e) Flow cytometry assessment of cell apoptosis by annexin-V/PI (d) and two representative groups after transient knockdown of FGFR1 in HUH-7 and HUH-7/R cells was statistically analyzed (e). (f)–(l) Western blotting analysis of p-FGFR1/FGFR1, cleaved caspase 3, cleaved caspase 9, Bax, and Bcl-2 was carried out in HUH-7 and HUH-7/R cells after transient knockdown of FGFR1 and combined treatment with sorafenib and DJ-1. NS: Not significant, ^∗^*P* < 0.05 versus DJ-1 group, and ^∗∗^*P* < 0.01 versus the vehicle or DJ-1 alone.

Cellular sensitivity to sorafenib (1, 2, 5, 10, 20, 50, 100, and 150 *μ*M) was measured by the CCK8 assay, and the IC_50_ was determined as 2.5 *μ*M for THLE-2 cells, 4 *μ*M for HepG2, and 3.5 *μ*M for HUH-7, respectively (Figure 6(a)). The sorafenib-resistant HUH-7 cell line (HUH-7/R) was constructed (Figure 6(b)), and the RI was 1.61. Moreover, the resistant characteristics were verified for the increased expression of P-gp and MRP1 (Figure 6(c)). Compared with HUH-7 cells, the apoptotic cells were decreased in the HUH-7/R group after sorafenib treatment, as well as in the condition treated with sorafenib and DJ-1. However, the apoptotic effect was enhanced in HUH-7/R cells after transient downregulation of FGFR1 after combined treatment with sorafenib and DJ-1, resembling the results observed following HUH-7 cell treatment with sorafenib and DJ-1 (Figures 6(d) and 6(e)). These results suggested that downregulation of FGFR1 could reverse HCC cell resistance to sorafenib to some extent. In addition, after transient downregulation of FGFR1 and combined treatment with sorafenib and DJ-1, the expression of proapoptotic markers, i.e., cleaved caspase 3, cleaved caspase 9, and Bax, was all increased (Figures 6(f)–6(l)), and the expression of the antiapoptotic marker (Bcl-2) was decreased (Figures 6(f)–6(l); Supplementary Figure 1). These results together implied that FGFR1 could affect survival of sorafenib-resistant HCC cells via regulating apoptosis-associated molecules.

### 3.6. Targeting FGFR1 Could Suppress Tumor Formation and Angiogenesis of the Sorafenib-Resistant HCC Cells *In Vivo*

**Figure 7:**
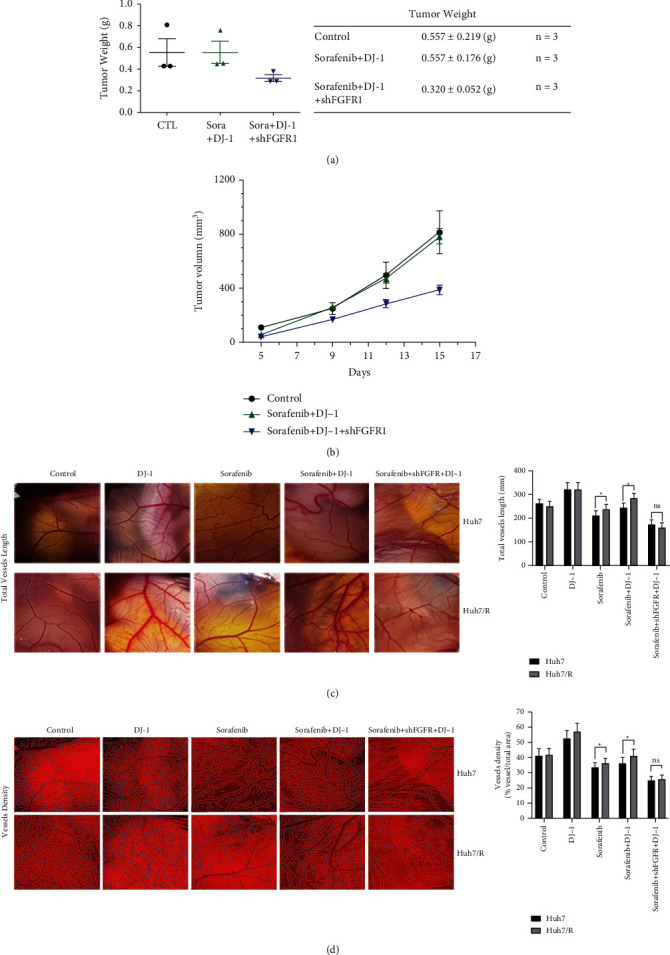
FGFR1 knockdown impairs tumor growth and angiogenesis of sorafenib-resistant HCC cells *in vivo.* (a and b) Pilot studies of tumor xenograft experiments were performed for HUH-7/R cells with 3 groups of treatment: i.e., vehicle control; sorafenib+DJ-1; and sorafenib+DJ-1+shFGFR1. 5 × 10^6^ cells per injection were injected subcutaneously into two flanks of nude mice (*n* = 3), and tumor weight (a) and tumor volume (b) were recorded. Tumor volume was checked twice to three times weekly using a digital caliper. (c and d) Tumor cell angiogenesis was examined in the chicken chorioallantoic membrane (CAM) model. Both HUH-7 and HUH-7/R cells were mixed with ECDHCC-1 cells at the ratio of 1 : 1 and then treated at different conditions: (1) cells+control; (2) cells+DJ-1; (3) cells+corafenib; (4) cells+sorafenib+DJ-1; and (5) cells+sorafenib+DJ-1+shFGFR1. In CAM model, total vessel length (c) and density (d) were measured at the end of the experiments.

To further confirm if FGFR1 plays a vital role in sorafenib resistance in HCC cells, we first chose HUH-7/R cell line as our model and treated HUH-7/R cells at different conditions, i.e., vehicle control, sorafenib, sorafenib plus DJ-1, and sorafenib plus DJ-1 and FGFR1 knockdown, where were then injected subcutaneously into immunodeficient mice (i.e., nude mice). In our pilot study, we found that FGFR1 knockdown showed the trend of inhibiting tumor growth of sorafenib-resistant cells on the condition of sorafenib and DJ-1 treatment (Figures 7(a)–7(c)). In support, we applied the chicken chorioallantoic membrane (CAM) model, which has been widely used as an in vivo tool to study tumor angiogenesis and metastasis [17, 18]. In our study, we used HUH-7 cells and HUH-7/R cells and divided them into 5 groups: (a) cells+control treatment; (b) cells+DJ-1; (c) cells+sorafenib; (d) cells+sorafenib+DJ-1; and (e) cells+sorafenib+DJ-1+shFGFR1. Compared to the control group, we found that (1) DJ-1 overexpression led to the increase of total vessel length and vessel density; (2) sorafenib inhibited angiogenesis of HUH-7 cells by shortening their vessel length, but not the HUH-7/R cells; and (3) FGFR1 knockdown dramatically inhibited angiogenesis of HUH-7/R cells with both sorafenib treatment and DJ-1 overexpression (Figures 7(d) and 7(e)).

At the end of this experiment, we harvested cells from tissues derived in CAM model and checked for their expression of the FGFR1 and apoptosis-associated molecules (Supplementary Figure 2). Consistently, we found that FGFR1 knockdown could increase the expression of proapoptotic molecules and inhibit the expression of antiapoptotic molecules (Supplementary Figure 2). These data, altogether, suggested that FGFR1 knockdown can reverse sorafenib resistance in HCC *in vivo*.

## 4. Discussion

In the present study, we isolated and purified DJ-1 secreted by HCC cells and found that DJ-1 could induce the phosphorylation of FGFR1 and promote angiogenesis and migration of ECDHCC-1 cells. The downstream mechanisms of DJ-1/FGFR1 involved the activation of the Akt/mTOR, ERK1/2, and STAT3 signaling pathways. Furthermore, knockdown of FGFR1 inhibited the tube formation and migration of ECDHCC-1 cells stimulated by DJ-1. It also displayed a significant proapoptotic effect comparable to that of sorafenib on nonresistant HCC cells in the presence of DJ-1, which may imply reversal of sorafenib resistance in HCC cells. These findings suggested that extracellular DJ-1 secreted by HCC cells might be a cross-talk regulator between tumor cells and vascular endothelial cells. The inhibition of DJ-1/FGFR1 signaling may be crucial to reverse the resistance of HCC to sorafenib, especially with tumors bearing high levels of DJ-1.

We have reported that DJ-1 expression was significantly upregulated in HCC, and the levels correlated with preoperative AFP, liver cirrhosis, vein invasion, differentiation, and Edmondson grade of HCC. Moreover, both tumor-free survival time and overall survival time in the DJ-1 high expression group were shorter than those in the low expression group. Hence, DJ-1 was proposed as an independent prognostic factor for overall survival of HCC patients [7]. Additionally, we verified that stable knockdown of DJ-1 decreased proliferation, adhesion, and invasion of HepG2 cells *in vitro* and inhibited the growth of HepG2-induced tumor *in vivo*, suggesting a crucial role for DJ-1 in the oncogenesis of HCC [8].

The curative treatment of resection or liver transplantation is usually applicable for patients diagnosed at an early-stage. For patients diagnosed at an advanced stage of HCC, sorafenib is the first-line choice of systemic therapy. However, almost all HCC patients at the late stages eventually fail in sorafenib treatment due to sorafenib resistance [19]. Our previous study suggested that DJ-1 shRNA effectively reversed the Adriamycin resistance of human breast cancer cells with a 2.68-fold increase in the sensitivity to the Adriamycin [20]. Meanwhile, FGFR, a receptor tyrosine kinase, has been identified as a critical regulator of vascular development, and the activation of FGF/FGFR signaling was considered as the driver of acquired resistance to sorafenib [11]. In the current study, we hypothesized DJ-1 as a cross-talk regulator between HCC cells and vascular endothelial cells. Interestingly, we found that DJ-1 could promote angiogenesis in HCC vascular endothelial cells via activation of FGFR1 in this study, suggesting an important role of DJ-1/FGFR1 signaling in the development of angiogenesis.

It has been reported that sorafenib suppresses tumor angiogenesis and proliferation by inhibiting the signals mediated by serine/threonine kinases, such as Raf/MEK/ERK1/2 cascade, as well as the signals mediated by receptor tyrosine kinases, such as FGFR, VEGFR, and PDGFR. These kinases are involved in cell proliferation, angiogenesis, and apoptosis [21]. In details, the analysis of *in vitro* experiments reported that there was a strong correlation between the inhibition of the Raf/MEK/ERK1/2 cascade and the anticlonogenic effect of sorafenib. Inhibition of the VEGFR accounts, at least in part, for the antiangiogenic effects of sorafenib [22]. Moreover, overexpression of FGFR1, the predominant FGFR in endothelial cells, stimulates endothelial cell proliferation [10]. In addition, other signaling pathways are implicated in the initiation and progression of HCC, such as PI3K/Akt/mTOR, JAK/STAT, and the Wnt/*β*-catenin cascade [23]. Results from previous studies suggested that DJ-1 induced phosphorylation of FGFR1 and activated the FAK and ERK1/2 signaling pathways and resulted in the stimulation of migration and capillary formation of vascular endothelium [9]. Furthermore, we have previously determined that the knockdown of DJ-1 inhibited human HepG2 cell growth and xenograft-induced tumor generation potentially through the Akt signaling pathway [8], and in the present study, we found that DJ-1 induced the activation of downstream Akt/mTOR, ERK1/2, and STAT3 in ECDHCC-1 cells via at least partially FGFR1, which suggested the important role of DJ-1/FGFR1 signaling pathway on the initiation, progression, proliferation, and angiogenesis in HCC vascular endothelium. Hence, DJ-1 antagonizes the therapeutic effect of sorafenib and may lead to therapy resistance. Our results are in the agreement with previous studies which showed that overexpression of DJ-1 can increase the drug resistance of cancer cells including pancreatic cancer [24], nonsmall cell lung cancer (NSCLC) [25], and leukemia [26].

The cytotoxic effect of sorafenib plays a crucial role in antitumor treatment. Generally, apoptosis is the major form of cytotoxicity and it is required for tumor regression and sustained clinical remissions [27–29]. Sorafenib downregulates the antiapoptotic molecules (such as Bcl-2) and increases the expression of proapoptotic molecules, such as Bax and p53-upregulated-modulator-of-apoptosis (PUMA) [29]. Conversely, the oncogenic effect of DJ-1 is mainly attributed to its antiapoptotic ability. In the present study, a sorafenib-resistant HCC cell line HUH-7/R was established. Treatment with DJ-1 inhibited the apoptotic effect of sorafenib in both HUH-7 and HUH-7/R cells. In addition, knockdown of FGFR1 could lead to a proapoptotic effect on HUH-7/R cell line after treatment of sorafenib and DJ-1, with significant upregulation of cleaved caspase 3/9 and Bax and an obvious downregulation of Bcl-2. Importantly, our pilot xenograft experiments showed the trend that FGFR1 knockdown could impair tumor growth of sorafenib-resistant HCC cells in the presence of DJ-1 treatment. Furthermore, our studies in the CAM model revealed that DJ-1 overexpression promoted angiogenesis of sorafenib-resistant HCC cells, but this phenotype could potentially be reversed by FGFR1 knockdown (Figure 7). Altogether, our results suggested that DJ-1/FGFR-1 signaling pathway contribute to sorafenib resistance in HCC, and downregulation of DJ-1/FGFR1 signaling might be a promising strategy to improve the curative effect of sorafenib, especially in those patients with high expression of DJ-1.

Nevertheless, our current studies have some limitations. For example, although our pilot xenograft studies displayed the trend of inhibiting tumorigenicity of sorafenib-resistant HCC cells via FGFR1 knockdown, a large cohort of mouse xenograft study may be needed to verify this phenotype. Also, we propose that DJ-1 is probably one of the factors secreted from culture medium of HCC cells, which may impact the biological features of endothelial cells. Future work involves of high-throughput screening is indispensable to uncover novel molecules that is potentially responsible for sorafenib resistance. Moreover, a genetically engineered model is required to specifically knock out DJ-1, using either DJ-1 conditional knockout mouse model [30] or establishing DJ-1-deleted sorafenib-resistant HCC cells via CRISPER, to provide the direct evidence that DJ-1 is vital in sorafenib resistance in HCC and DJ-1/FGFR1 signaling is a potent target. In addition, detailed mechanisms underlying sorafenib resistance via DJ-1/FGFR1 signaling shall be uncovered in the future directions.

## 5. Conclusion

In summary, the present study hinted that HCC cells may interact with vascular endothelial cells via DJ-1. Moreover, FGFG1 downregulation can impair angiogenesis and tumor growth of sorafenib-resistant HCC cells on the condition of DJ-1. Altogether, our data provide the evidence that DJ-1 and FGFR-1 signaling may be a potential target for treating patients with sorafenib resistance.

## Data Availability

The original contributions presented in the study are included in the article Material. Further inquiries can be directed to the corresponding author.

## Supplementary Materials

Supplementary Materials Figure 1 FGFR1 knockdown increased the expression of proapoptotic markers but impairs the expression of antiapoptotic markers *in vitro.* Western blotting analysis of p-FGFR1/FGFR1, cleaved caspase 3, cleaved caspase 9, Bax, and Bcl-2 was performed for HUH-7/R cells after treatment of DJ-1 and FGFR1 in the presence of sorafenib. Downregulation of FGFR1 led to elevated level of proapoptotic molecules, including caspase 3, cleaved caspase 9, and Bax, but dampened level of antiapoptotic molecule, such as BCL-2. Supplementary Figure 2: knocking down of FGFR1 increased the expression of proapoptotic markers but impairs the expression of antiapoptotic markers in the CAM model. In the CAM model, tissues were harvested at the end of the experiments from each group, which were then checked for the expression of p-FGFR1/FGFR1, cleaved caspase 3, cleaved caspase 9, Bax, and Bcl-2 via western blotting analysis. Consistently, FGFR1 knockdown could induce the expression of proapoptotic molecules (caspase 3, cleaved caspase 9, and Bax) and decrease level of antiapoptotic molecule (BCL-2).
