# Supplementary figures and images for: DJ-1/FGFR-1 Signaling Pathway Contributes to Sorafenib Resistance in Hepatocellular Carcinoma
Source: Oxid Med Cell Longev. 2022 Jun 20;2022:2543220. doi: 10.1155/2022/2543220 (PMC9236769; doi:10.1155/2022/2543220)

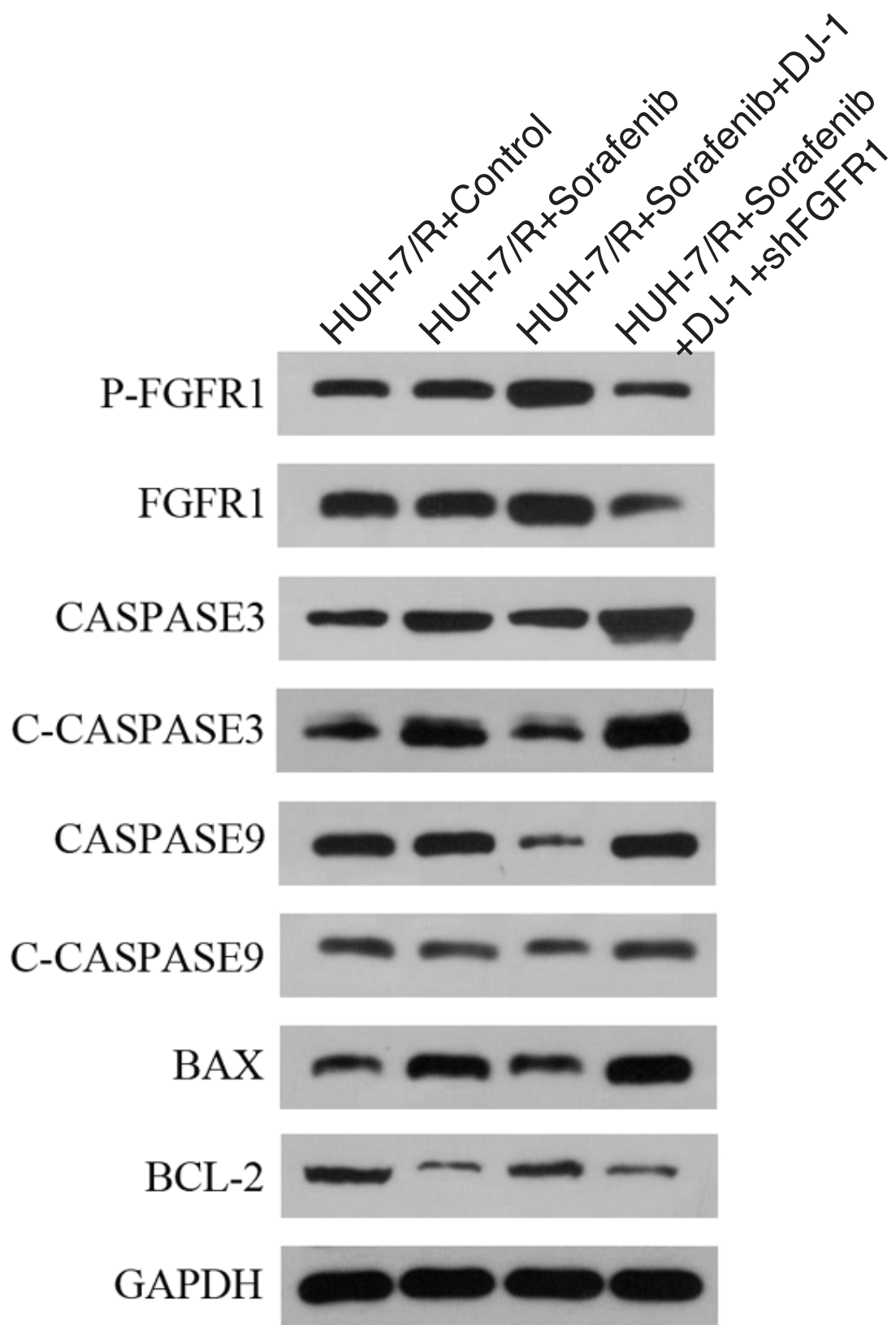

Chen et al Supplementary Figure 1

Supplement: Supplementary Materials — Figure 1 FGFR1 knockdown increased the expression of proapoptotic markers but impairs the expression of antiapoptotic markers in vitro. Western blotting analysis of p-FGFR1/FGFR1, cleaved caspase 3, cleaved caspase 9, Bax, and Bcl-2 was performed for HUH-7/R cells after treatment of DJ-1 and FGFR1 in the presence of sorafenib. Downregulation of FGFR1 led to elevated level of proapoptotic molecules, including caspase 3, cleaved caspase 9, and Bax, but dampened level of antiapoptotic molecule, such as BCL-2. Supplementary Figure 2: knocking down of FGFR1 increased the expression of proapoptotic markers but impairs the expression of antiapoptotic markers in the CAM model. In the CAM model, tissues were harvested at the end of the experiments from each group, which were then checked for the expression of p-FGFR1/FGFR1, cleaved caspase 3, cleaved caspase 9, Bax, and Bcl-2 via western blotting analysis. Consistently, FGFR1 knockdown could induce the expression of proapoptotic molecules (caspase 3, cleaved caspase 9, and Bax) and decrease level of antiapoptotic molecule (BCL-2). [file 2543220.f1.zip › Supplementary Figure 1.pdf]

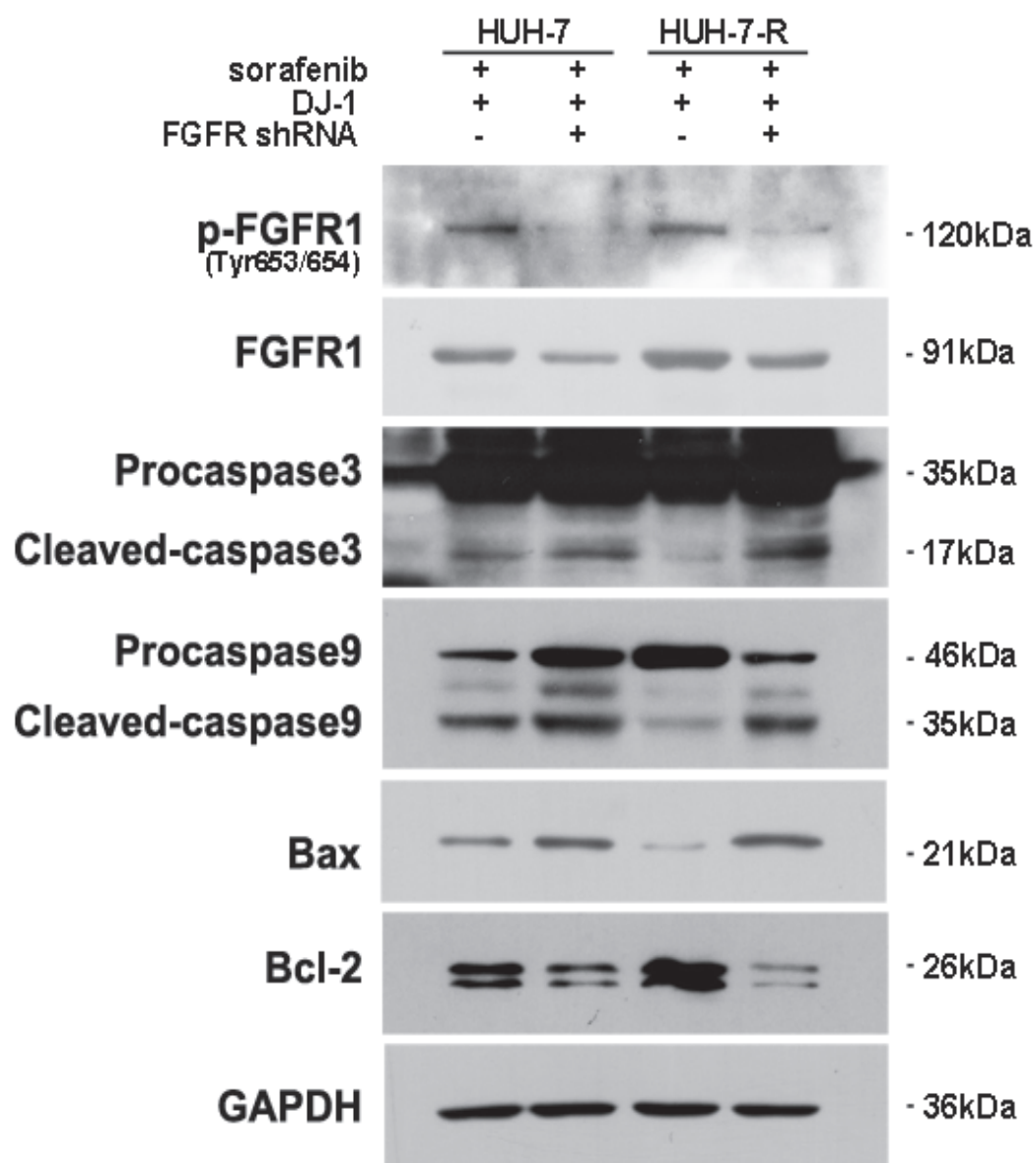

Chen et al Supplementary Figure 2

Supplement: Supplementary Materials — Figure 1 FGFR1 knockdown increased the expression of proapoptotic markers but impairs the expression of antiapoptotic markers in vitro. Western blotting analysis of p-FGFR1/FGFR1, cleaved caspase 3, cleaved caspase 9, Bax, and Bcl-2 was performed for HUH-7/R cells after treatment of DJ-1 and FGFR1 in the presence of sorafenib. Downregulation of FGFR1 led to elevated level of proapoptotic molecules, including caspase 3, cleaved caspase 9, and Bax, but dampened level of antiapoptotic molecule, such as BCL-2. Supplementary Figure 2: knocking down of FGFR1 increased the expression of proapoptotic markers but impairs the expression of antiapoptotic markers in the CAM model. In the CAM model, tissues were harvested at the end of the experiments from each group, which were then checked for the expression of p-FGFR1/FGFR1, cleaved caspase 3, cleaved caspase 9, Bax, and Bcl-2 via western blotting analysis. Consistently, FGFR1 knockdown could induce the expression of proapoptotic molecules (caspase 3, cleaved caspase 9, and Bax) and decrease level of antiapoptotic molecule (BCL-2). [file 2543220.f1.zip › Supplementary Figure 2.pdf]
